# Manipulation of Costimulatory Molecules by Intracellular Pathogens: Veni, Vidi, Vici!!

**DOI:** 10.1371/journal.ppat.1002676

**Published:** 2012-06-14

**Authors:** Nargis Khan, Uthaman Gowthaman, Susanta Pahari, Javed N. Agrewala

**Affiliations:** CSIR-Institute of Microbial Technology, Chandigarh, India; University of Alberta, Canada

## Abstract

Some of the most successful pathogens of human, such as *Mycobacterium tuberculosis (Mtb)*, HIV, and *Leishmania donovani* not only establish chronic infections but also remain a grave global threat. These pathogens have developed innovative strategies to evade immune responses such as antigenic shift and drift, interference with antigen processing/presentation, subversion of phagocytosis, induction of immune regulatory pathways, and manipulation of the costimulatory molecules. Costimulatory molecules expressed on the surface of various cells play a decisive role in the initiation and sustenance of immunity. Exploitation of the “code of conduct” of costimulation pathways provides evolutionary incentive to the pathogens and thereby abates the functioning of the immune system. Here we review how *Mtb*, HIV, *Leishmania sp.*, and other pathogens manipulate costimulatory molecules to establish chronic infection. Impairment by pathogens in the signaling events delivered by costimulatory molecules may be responsible for defective T-cell responses; consequently organisms grow unhindered in the host cells. This review summarizes the convergent devices that pathogens employ to tune and tame the immune system using costimulatory molecules. Studying host-pathogen interaction in context with costimulatory signals may unveil the molecular mechanism that will help in understanding the survival/death of the pathogens. We emphasize that the very same pathways can potentially be exploited to develop immunotherapeutic strategies to eliminate intracellular pathogens.

## Introduction

The immune system is highly evolved to combat and eliminate pathogens. However, some pathogens can successfully subvert the host immune system to establish their intracellular survival via strategies such as the disguise or sequestration of antigens, molecular mimicry, immunosuppression, circumvention of complements and cytokines cascade, blockade of antigen presentation, escape from apoptosis and autophagy, and modulation of costimulatory signals [1].

T cells and antigen-presenting cells (APCs) play crucial roles in eliminating intracellular pathogens. The optimal activation of naive T cells is achieved by occupancy of T-cell receptor (TCR) by the peptide-MHC complex displayed on the surface of APCs, delivery of costimulatory signals, and the presence of proinflammatory cytokines [2]. The expression of costimulatory molecules on APCs is critical in shaping the extent and nature of the immune response. Thus, an encounter of T cells with peptide-MHC can result in two discrete events: (a) T-cell proliferation and differentiation into effector cells; (b) or anergy or apoptosis. The question of which of these outcomes transpires is determined by the delivery of appropriate costimulatory signals [3]. An array of costimulatory molecules is displayed on the surface of APCs (CD80/B7-1, CD86/B7-2, CD83, CD40, PDL-1, DC-SIGN, 4-1BBL, etc.) and T cells (CD28, CTLA-4, CD40L, PD-1, OX40, 4-1BB, etc). The level of the expression of the costimulatory molecules may play an important role during the course of acute disease and its remission or relapse. Hence, modulation of these molecules by pathogens can help them to establish their existence in the host.

Here we elaborate the mechanism by which pathogens such as *Mtb*, HIV, and *Leishmania sp.*, employ molecules viz. CD28, CD40, CD40L, CD80, CD86, CTLA-4, PD1, PDL-1, etc., to break the code of costimulation to establish chronic infection. The organisms in consideration are representatives of successful pathogens from the class of bacteria, virus, and parasites. It appears that these pathogens adopt a common evolutionarily convergent mechanism to evade host immune reaction. This review provides insights into the mechanism by which pathogens suppress host immunity by modulating the expression of costimulatory molecules. We also suggest avenues of therapeutic intervention by exploiting costimulatory pathways for treating infections.

## The Paradigm Shift in the Biology of Costimulation

**Figure 1:**
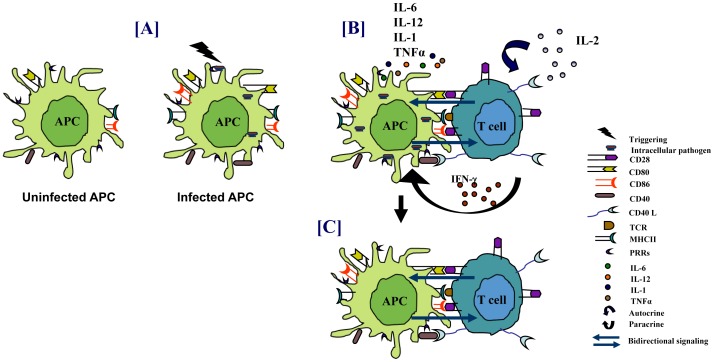
Immune response against intracellular pathogens. (A) PRRs of APCs sense pathogens that result in the activation of APCs. (B) This leads to enhanced antigen presentation, upregulation of costimulatory molecules, and secretion of proinflammatory cytokines that promote the activation of T cells. The activated T cells help in elimination of the pathogens. (C) Engagement of costimulatory molecules on APCs by T cells also results in “bidirectional signaling” that activates APCs to restrict the growth of pathogens.

The unilateral “help to T-cell” lymphocentric paradigm of costimulatory pathways has currently evolved into a bilateral signaling model that influences the activity of both T cells and APCs during their interaction (Figure 1) [4], [5]. Costimulatory molecules of CD80/CD28, tumor necrosis factor (TNF)/TNFR, and TIM superfamilies have unmasked the plethora of the possible ligand-receptor interactions that has expanded the understanding of regulation of the immune responses mediated by APCs and T cells. For example, a positive regulator like CD40L (on T cells) when associated with CD40 (on APCs), not only activates T cells but also results in the activation of dendritic cells (DCs); a process that is popularly called “T-cell licensing” [6]. Similarly, ligation of CD28 with CD80 and CD86 is known to induce the secretion of interleukin-6 (IL-6) and interferon-γ (IFN-γ) by DCs and activation, proliferation, and differentiation of B cells [5], [7], [8]. It is reported that 4-1BBL expressed on DCs, binds to 4-1BB on T cells, to bolster DCs help to T cells [9]. Many reports have highlighted the inhibitory roles of CTLA-4 (CD152) and PD-1 (expressed on T cells) with ligands CD80/CD86 and PDL-1/PDL-2 (on APCs), respectively [10], [11]. It clearly suggests that costimulation not only amplifies the magnitude of the activation of T cells and APCs, but fine tunes the immune response as well, thereby controlling the hyperactivation.

## Modulation of Costimulatory Molecules by Bacteria

Intracellular pathogens like *Mtb*, *Mycobacteria avium*, *M. leprae, Salmonella typhi* (*S. typhi*), *Helicobacter pylori* (*H. pylori*), etc., infect both macrophages and DCs. These cells recognize bacterial components known as pathogen-associated molecular patterns (PAMPs) through their pathogen recognition receptors (PRRs). This triggers innate immunity that initiates antimicrobial defense mechanisms involving autophagy, apoptosis, release of antimicrobial compounds like IFN-γ and TNF-α, etc. [12]. It is followed by activation of adaptive immunity. The adaptive immune response, personified by specificity and memory, involves the clonal selection of B cells and T cells that confer humoral immunity (HI) and cell-mediated immunity (CMI) to pathogens, respectively. Pathogen-specific Th1 cells release cytokines, especially IFN-γ and TNF-α, which plays an imperative role in activating infected macrophages and restraining the microbial growth [13]. Further, ligation of CD80/CD86 with CD28 is essential for preventing apoptosis in T cells [7], [14]. Moreover, CD40/CD40L interaction results in efficient IL-12 production together with an upregulation of costimulatory molecules, in addition to enhanced antigen presentation by APCs thereby leading to a boosted T-cell response (Figure 1) [6], [15]. Hence, interference in the down-modulation of any of these pathways by the pathogens would be detrimental to the host.

*Mtb* is one of the most successful pathogens in the history of mankind. There are numerous reports indicating the role of mycobacteria in downregulating the expression of CD80, CD86, and CD40 on APCs [16], [17]. A recent study showed, albeit for BCG, that MHC-II, CD80, CD86, and CD40 are down-tuned during chronic phase of infection [16]. In such studies, it is important to delineate the expression of these molecules on infected versus non-infected cells, as by-stander inflammation could interfere in the interpretation of results. An elegant study demonstrated that the expression of costimulatory molecules and MHC are downregulated in macrophages infected with fluorescent reporter bacteria [16], [18]. In contrast, others suggested the augmentation of costimulatory molecules upon infection [19]. This discrepancy may be primarily dependent on the strain, system, or time-point of the study.

**Figure 2:**
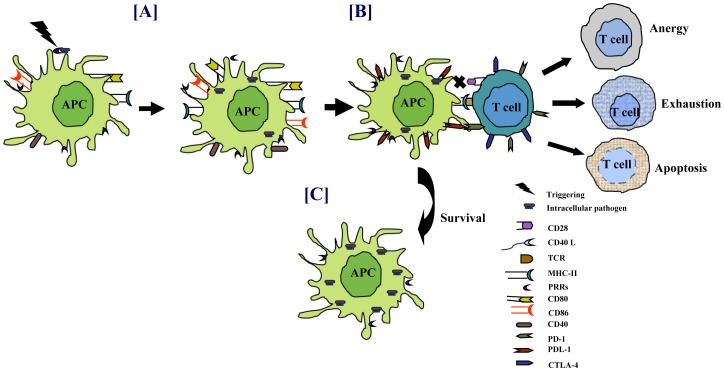
Pathogens modulate the expression of costimulatory molecules for their survival. Sensing of pathogens through PRRs triggers the activation of APCs. (A) Costimulatory molecules, which act as the second signal for T-cell activation, are upregulated on infected cells. Persistence of intracellular pathogens modulates the expression of costimulatory molecules, such as downregulation of CD40/CD80/CD86 and upregulation of PDL-1 on infected APCs. Similarly, retarding the exhibition of CD28/CD40L augments PD-1/CTLA-4 on T cells. (B) Interaction of T cells with the infected APCs impairs the function of T cells by inducing anergy, apoptosis, or exhaustion. (C) Lack of T-cell help impedes the activity of APCs, eventually enhancing the survival of pathogens.

Downregulation of CD80/CD86 or upregulation of CTLA-4 by bacteria on APCs may induce anergy/apoptosis of interacting T cells [20], [21]. Defect in this signaling pathway is known to paralyze the release of IL-2, which may compromise the generation of T-cell memory [22]. Impediment in CD28 signaling interferes in IFN-γ production and hence promotes the survival of pathogens. It has been reported that *M. leprae* obstructs CD28/B7 signaling pathway for rendering antigen-specific T cell unresponsive in lepromatous leprosy patients [21]. Recently, the importance of CD80/CD86 in controlling mycobacterial infection has been demonstrated in CD80/CD86 double knockout mice [23]. The down-modulation of CD80/CD86 in chronic phase of the infection suggests that mycobacteria may actively exploit this pathway to anergize the T cells (Figure 2). Protective CMI is always associated with the release of chemokines and migration of immune cells to the site of infection. *Mtb* impair chemokines secretion by interfering with the CD28-B7 signaling pathway thereby obstructing the surveillance of immune cells and enhancing the propagation of the bacterium [24], [25]. It has been shown that the most abundant cell wall lipid trehalose 6, 6′-dimycolate (TDM) of *Mtb* and MTSA-10 inhibit the expression of costimulatory molecules on the surface of the macrophages [26], [27]. Similarly, CD40-CD40L interaction is very important in mediating efficient protection against mycobacteria [28]. Indeed, it has been shown in vitro and in lepromatous patients that CD40 is downregulated by *M. leprae* [29], [30]. There is indirect evidence indicating the involvement of *Mtb* manipulating CD40/CD40L expression. In humans, CD40L expression on Th1 cells of tuberculosis (TB) patients has been correlated with the intensity of IFN-γ secretion [31]. However, CD40 but not CD40L knockout mice are susceptible to *Mtb* infection [32], [33]. It is reported that the heat shock protein 70 (HSP70) of *Mtb* acts as an alternate ligand for CD40. Corroboratively, over-expression of HSP70 interferes with long-time persistence of *Mtb* and allows its clearance [34]. Interestingly, in chronic mycobacterial infections, CD40 is suppressed on infected cells [16]. Therefore, it is intriguing to speculate that in the chronic phase of infection, mycobacteria may hamper CD40 expression or manipulate CD40L signaling through binding with HSP70 instead of CD40L in order to evade host defense mechanisms.

Mycobacteria can upregulate the expression of PD-1 and its ligands PDL-1/PDL-2 [35]. Several studies have shown that T cells and natural killer (NK) cells from TB patients have increased PD-1 expression [36], [37]. Blockade of PD-1 interaction with PDL-1/PDL-2 enhances immune response. These observations suggest that mycobacteria may exploit PD-1 and PDL-1/PDL-2 pathways to dampen the host immune responses. In contrast, some studies have suggested that these pathways may be involved in controlling exuberant T-cell responses in TB and hence may be beneficial for both the host and the pathogen [36], [38], [39]. *Mtb* retards the development and maturation of monocyte-derived DCs to limit the immune response [40]. These immature DCs have a tolerogenic effect *in vivo* that can result in the generation of regulatory T cells (Tregs). Tregs can restrain the proliferation of naive and memory T cells, thereby suppressing pre-existing T-cell immunity [41], [42].

Besides mycobacteria, many other pathogens can exploit CD80/CD86-CD28/CTLA-4 pathways for their persistence (Table 1). *H. pylori* causes chronic infection in the gut resulting in peptic ulcers. Further, it is known to induce the expression of CTLA-4, resulting in the anergy of T cells and poor clearance of the bacteria [43]. *Yersinia pseudotuberculosis* decreases CD86 expression on B cells and impedes the function of both B cells and T cells [44]. *S. typhi* is known to suppress ICAM-1 and as a consequence reduces the antigen uptake by APCs and inadequate T-cell response [45], [46]. *H. pylori* diminishes the expression of CD40L on T cells and therefore employs CD40/CD40L pathway for its survival. Furthermore, it upregulates PDL-1 expression on gastric epithelial cells and inhibits the activation of T cells recruited to gastric mucosa [47]. In addition, it has been reported that PDL-1 upregulation not only blocks T-cell proliferation and IL-2 secretion, but also promotes the development of Tregs [48]. *Bordetella pertussis* and *B. bronchiseptica* decrease the manifestation of CD40 and ICAM-1 on DCs, and subsequently, render DCs tolerogenic and promote chiefly Tregs but not Th1 cells [49], [50]. Hence, costimulatory molecules could serve as attractive targets for bacteria to modulate in order to prolong their own survival.

**Table 1:** Exploitation of costimulatory molecules by intracellular pathogens.

Intracellular Pathogens	Costimulatory Molecules	Loss of Function	References
**Bacteria**			
***M. tuberculosis***	CD80↓, CD86↓, CD40↓, PDL-1/PDL-2↑, PD-1↑	Hampers effective T-cell activation. Induces anergy or apoptosis in T cells, paralyzes IL-2 and chemokines secretion, and inhibits NK cell function.	[16], [20], [35], [37]
***M. leprae***	CD80↓, CD28↓	Blockade of IL-12 secretion, defective T-cell response	[21]
***S. typhimurium***	ICAM-1↓	Impedes antigen uptake ability of APCs	[45], [46]
***B. anthracis***	CD40↓, CD80↓, CD86↓	Impairment of antigen specific B-cell and T-cell immunity, suppresses the function of DCs	[104], [105]
***H. pyroli***	PDL-1↑, CTLA-4↑	Exhaustion of DCs, obstructs cytokines secretion, induces anergy in T cells	[43], [106]
***B. bronchiseptica***	CD40↓	Hampers maturation of DCs	[49]
***B. pertussis***	CD40↓, ICAM↓	Promotes differentiation of Tregs	[50]
**Viruses**			
**HIV**	PD-1↑, PDL-1↑, CTLA-4↑, CD80↓, CD86↓, CD33↓, CD40L↓, 4-1BB↓, OX40↓	Blocks IL-2 but augments IL-10 secretion, induces exhaustion of T cells, defective CTLs response, and hampers antigen uptake ability of APCs	[58], [60], [69], [75]
**HBV**	PDL-1↑, PD-1↑	Induces IL-10 secretion, enhances apoptosis and anergy in T cells	[79]
**HCV**	CD83↓, CD86↓	Reduces stimulatory capacity of DCs	[72]
**Measles**	CD40↓, CD80↓, CD86↓, CD25↓, CD83↓, CD69↓	Abnormal DCs differentiation, improper CD8 T-cell proliferation	[70]
**Herpes simplex virus**	ICAM-1↓	Blocks APCs T-cell communication	[71], [73]
**Protozoans**			
***L. donaovani***	CD80↓	Inefficient T-cell response	[81], [107]
***T. gonodii***	CD80↓	Inhibits T-cell stimulatory activity	[82]

**↑:** ,upregulation;

**↓:** , downregulation.

## Modulation of Costimulatory Molecules by Viruses

To counter viral infestation, the vertebrate immune system has evolved complex antiviral innate and adaptive immune mechanisms. Some of the spectacular examples to limit viral replication by the host cells are the production of interferons, NK cell-mediated lysis, and apoptosis of the infected cells [51], [52]. Adaptive antiviral immunity relies greatly on the lysis of infected cells by cytotoxic CD8^+^ T cells and neutralizing antibodies secreted by B cells.

HIV is a retrovirus that is responsible for 1.9 million deaths annually [53]. It predominantly infects CD4^+^ T cells. Further, it invades DCs, monocytes, and macrophages expressing CD4 and one of its coreceptors CCR5 or CXCR4 [54]. Additionally, HIV can bind to DC-SIGN and mannose receptors [55]. However, the virus preferentially replicates in the activated HIV-specific CD4^+^ T cells [56]. Indeed, excessive loss of CD4^+^ T cells is the hallmark of HIV infection [57]. Like many other intracellular pathogens, HIV efficiently exploits the costimulatory molecules to override the immune responses. Its infection is associated with decreased expression of CD40L on CD4^+^ T cells [58]. Upon activation, CD4^+^ T cells from individuals with progressive disease show very little upregulation of CD40L, which corroborates with their inability to help APCs and failure to induce IL-12 in DCs [59]. Further, CD40-CD40L interaction has been demonstrated to be important in engendering a robust HIV-specific CD8^+^ T-cell response. Furthermore, HIV interferes in the CD40 signaling pathway in B cells and hinders T cell help, thereby impairing the secretion of IgG and IgA antibodies [60], [61]. AIDS patients suffer from a defective humoral immunity, which may be due to loss of T-cell function, or B-cell intrinsic defects [62], [63]. HIV upregulates Fas and FasL (members of TNF superfamily) on CD8^+^ T cells and APCs, respectively, which leads to the apoptosis of the interacting CD8^+^ T cells [64], [65]. Thus, in HIV infections, the hunter becomes the hunted!

During viral infections, continual expression of CD80/CD86 on DCs is decisive to maintain the effector function of CD8^+^ T cells [66]. Intriguingly, HIV downregulates the expression of CD80/CD86 and their ligand CD28 on infected APCs and T cells, respectively [67], [68]. The expression of costimulatory molecules such as 4-1BBL, CD70, OX40, and OX40L is affected during HIV-1 infection [9], [69]. Measles, herpes, and hepatitis C viruses (HCV) retard the expression of CD80, CD86, CD25, CD83, and CD40 that leads to poor CD8^+^ T-cell priming [70]–[72]. In addition, Herpes virus suppresses ICAM-1 on APCs, thereby obstructing immunological synapse with T cells [73].

Chronic viral infections are associated with loss of function in T cells, a phenomenon popularly known as T-cell exhaustion. Exhausted T cells highly express PD-1 and have poor effector function [74]. PD-1 expression on HIV-specific T cells is associated with T-cell exhaustion and disease progression [75]. PD-1 is known to make CD8^+^ T cells more susceptible to Fas-mediated lysis [76]. Signaling through PD-1 can suppress IL-2 secretion by CD8^+^ T cells. IL-2 is known to rescue T cells from anergy and boost the memory response [77]. In addition, upregulation of PDL-1 on APCs during HIV or hepatitis B virus (HBV) infection supports the survival of pathogens (Table 1) [78], [79]. In essence, viruses can exploit costimulatory molecules in restraining the function of both T cells and APCs.

## Modulation of the Expression of Costimulatory Molecules by Intracellular Protozoan Parasites

T cells play a significant role in controlling the protozoa-inflicted diseases like malaria, visceral leishmaniasis, and trypanosomiasis. Humoral responses play a less important role. However, complement-mediated killing or opsonization is responsible for control of pathogen during the intermittent extracellular phase of its replication cycle within the vertebrate host [80].

Parasites like *Leishmania donovoni*, *L. chagasi*, *Toxoplasma gonodii* (*T. gonodii*), *T. cruzi*, and *Plasmodium falciparum* can manipulate the costimulatory molecules for evading immune system. *L. donovani* and *L. chagasi* infect macrophages and are reported to downregulate both CD80 and ICAM-1 [21], [81]. Such APCs fail to optimally activate T cells. *T. gondii* and *T. cruzi*, selectively dampen the exhibition of CD80 and CD28, respectively to impair the function of T cells [82]. CD40-CD40L signaling is crucial for Th1 immunity against *L. major*, because CD40L/CD40 knockout mice sparsely secrete IL-12, favoring Th2-biased response [83], [84]. Interestingly, *L. major* can differentially modulate the expression of CD40 and thus anergizes T cells and promotes Tregs population [83]. *Plasmodium* interferes in the signaling mechanism of CD40 in DCs [85]. CD40 deficient mice succumb to plasmodium infection. This signifies that this pathway is detrimental in providing sterilizing immunity to parasites, as it activates APCs and protective Th1 responses. In conclusion, parasites can efficiently empower the immune system by dampening the expression of costimulatory molecules.

## Mechanism Involved in Immunomodulation of Costimulatory Molecules

Induction of the expression of costimulatory molecules on exposure to various inflammatory cytokines (IL-6, IL-12, TNF-α, IFN-γ) or PAMPS/DAMPs is regulated by transcription factors such as NF-κB, IRF-3, AP-1, NFAT [86], [87]. Activation of these transcription factors is tightly regulated by various kinases or phosphatases that include mitogen-activated protein kinases (MAPKs), TNF receptor-associated factor proteins (TRAF), IL-1 receptor-associated kinase 4 (IRAK4), phosphoinositide 3-kinase (PI3K), and Janus-kinase. For example, triggering of TLR-4 with lipopolysaccharides (LPS) elicits pathways dependent on myeloid differentiation primary response gene 88 (MyD88) and TIR-domain-containing adapter-inducing interferon-β (TRIF). Both the molecules induce downstream signaling to activate MAPKs, NF-κB, and IRF family proteins, which are responsible for the enhanced expression of CD40 and CD86 [86], [88]. Further, T-cell interaction with APC involving TCR and costimulatory molecules activates a plethora of downstream signaling molecules, leading to the induction of the expression of CD40L, PD-1, and CD28 [89], [90].

Intracellular pathogens utilize an array of mechanisms to manipulate costimulatory molecules. Upon infection, pathogens trigger IL-10 secretion by inhibiting p38MAP kinases and promoting ERK phosphorylation [91]. IL-10 blocks the degradation of IκB-α that inhibits the NF-κB activation eventually inhibiting the expression of costimulatory molecules [92]. Interference in TLRs signaling by the intracellular pathogens is considered to be a foremost event in the suppression of costimulatory molecules. Mannosylated lipoarabinomannan (ManLAM) of *Mtb* binds to DC-SIGN and compromises the LPS-induced activation of DCs by interfering in the TLRs' signalling [93]. Binding of *Mtb* early secreted antigenic target protein 6 (ESAT-6) to TLR-2 activates AKT and prevents interaction between the MyD88 and IRAK4, consequently abrogating NF-κB activation that suppresses CD80 expression [94]. Similarly, HIV upregulates PDL-1 on DCs and monocytes by attenuating the signaling of TLR-7 and TLR-8 [95]. In addition, HIV-mediated PI3K activation upregulates PDL-1 on APCs and thus suppresses the activation of HIV-specific CD8^+^ T cells [96]. Rapid endocytosis of costimulatory molecules upon infection is suggested as one of the mechanisms to interrupt the function of APCs. For instance, Nef protein of HIV modulates the actin-dependent trafficking mechanism to remove CD80/CD86 from the monocytes surface, making them inefficient to activate T cells [97].

TCR signaling is central for the optimum expression of costimulatory molecules and effector function of T cells. Therefore, it may serve as an important target for pathogens to paralyze T-cell activity [98]. *Mtb* glycolipids interfere with TCR signaling and block the activation of CD4^+^ T cells [99]. HIV gp120 interacts with CD4 (a coreceptor for TCR) that inhibits intracellular signal transduction through TCR, leading to decreased hydrolysis of polyphosphoinositide (PI), Ca^2+^ influx, activation of protein kinase C (PKC), and eventually failure of NFAT translocation. These events culminate in the inhibition of CD40L expression. Decline of CD40L on T cells abrogates the bidirectional signaling and reduces the exhibition of CD80 on APCs [100]. In contrast, Nef activates NFAT and stimulates IL-2 release to overcome the exogenous requirement of IL-2 to promote T-cell proliferation, consequently disseminating the infection [101]. However, Nef retards CD28 and CD4 expression, thereby making T cells incapable of promoting CMI [98]. Corroborative with these observations, Nef-mutated HIV is unable to cause persistent infection. Similarly, core protein of HCV inhibits TCR signaling and upregulates PD-1 [102]. The above-mentioned mechanisms imply that pathogens can effectively utilize various costimulatory pathways to subvert immune response to persist in the host.

## Therapeutic Implications

The noncompliance with the relatively high dose and extended therapeutic regime reduces the effectiveness of current drugs, leading to global emergence of MDR/XDR/TDR pathogenic strains. Interestingly, costimulatory molecules have been suggested for therapeutic intervention to treat lymphoma patients [4], [103]. To develop alternative or adjunct (with drugs) therapies, an intensive effort has been undertaken in last decade to understand how intracellular pathogens exploit costimulatory molecules, which are the tour de force of the immune system [27], [31], [68], [78]. The potent role of costimulatory molecules is aptly established in the optimum activation of T cells and APCs; the cells that play a cardinal role in curbing the infections. Hence, immunotherapy involving costimulatory molecules can be a breakthrough strategy to treat various diseases, minimizing side effects inflicted by drug therapies and in restricting the emergence of drug resistance.
